# Low Subthreshold Slope AlGaN/GaN MOS-HEMT with Spike-Annealed HfO_2_ Gate Dielectric

**DOI:** 10.3390/mi12121441

**Published:** 2021-11-25

**Authors:** Min Jae Yeom, Jeong Yong Yang, Chan Ho Lee, Junseok Heo, Roy Byung Kyu Chung, Geonwook Yoo

**Affiliations:** 1 School of Electronic Engineering, Soongsil University, Seoul 06938, Korea; duaalswo94@gmail.com (M.J.Y.); skholda94@gmail.com (J.Y.Y.); yahco61@gmail.com (C.H.L.); 2 Department of Electrical and Computer Engineering, Ajou University, Suwon 16499, Korea; jsheo@ajou.ac.kr; 3 Department of Electronic Materials Science and Engineering, Kyungpook National University, Daegu 41566, Korea

**Keywords:** MOS-HEMT, ALD HfO_2_, Spike annealing, ferroelectric

## Abstract

AlGaN/GaN metal-oxide semiconductor high electron mobility transistors (MOS-HEMTs) with undoped ferroelectric HfO_2_ have been investigated. Annealing is often a critical step for improving the quality of as-deposited amorphous gate oxides. Thermal treatment of HfO_2_ gate dielectric, however, is known to degrade the oxide/nitride interface due to the formation of Ga-containing oxide. In this work, the undoped HfO_2_ gate dielectric was spike-annealed at 600 °C after the film was deposited by atomic layer deposition to improve the ferroelectricity without degrading the interface. As a result, the subthreshold slope of AlGaN/GaN MOS-HEMTs close to 60 mV/dec and on/off ratio>10^9^ were achieved. These results suggest optimizing the HfO_2_/nitride interface can be a critical step towards a low-loss high-power switching device.

## 1. Introduction

III-Nitrides (InN, GaN, and AlN) are well-known for their intrinsic spontaneous polarization and piezo-electricity. Furthermore, AlN and GaN exhibit large bandgap energy of 6.2 and 3.4 eV, respectively. Owing to these excellent material properties, AlGaN/GaN-based HEMTs have been extensively studied and are being commercialized for high-frequency and high-power applications [1,2,3]. Along with the vast improvement of material quality of nitrides, there have been significant improvements in device design to enhance the performance and reliability of HEMTs throughout the years [4,5]. To reduce the gate leakage and drain current collapse, for example, MOS-HEMTs have been developed with various high-k dielectrics such as Al_2_O_3_, Y_2_O_3_, and HfO_2_ [6,7,8]. HfO_2_ is a very promising gate dielectric for HEMTs due to its large bandgap (5.3–5.8 eV) and high dielectric constant (20–25).

HfO_2_ is especially interesting as a gate dielectric because of its ferroelectricity [9]. Ferroelectric gate can lead to a host of new and novel devices such as nonvolatile memory, radio-frequency, and neuromorphic devices [10,11]. When combined with AlGaN/GaN HEMTs, the reconfigurable polarization of HfO_2_ means polarization engineering for 2-dimensional electron gas (2DEG) channel. One of the advantages of polarization engineering is the ability to modulate the carrier density of 2DEG to lower the subthreshold slope (*SS*) and therefore reduce switching power loss. A large threshold voltage tuning (range: 2.8 V) and reduction of *SS* have been demonstrated with Hf_0_._5_Zr_0_._5_O_2_-based ferroelectric gate HEMTs recently [12]. While very promising, the effectiveness of a HfO_2_-based ferroelectric gate for HEMTs remains somewhat elusive as the reported average values of *SS* were still relatively large (160–180 mV/dec). Furthermore, it has been reported the interface of HfO_2_/GaN prone to the formation of unfavorable Ga–O bonds, which could potentially become interfacial traps [13].

In this work, AlGaN/GaN-based MOS-HEMTs with *SS* near 60 mV/dec and an on/off ratio of >10^9^ have been demonstrated. Undoped HfO_2_ was deposited by ALD as a gate dielectric. After spike-annealing, the ferroelectric property of HfO_2_ was assessed via high-resolution transmission electron microscopy (HR-TEM) and polarization voltage (P-V) PUND (Positive Up Negative Down) measurements on the fabricated HfO_2_/AlGaN MOSCAP. Due to the atomically abrupt HfO_2_/nitride interface and the spike annealing-induced orthorhombic phase in HfO_2_ film, the counterclockwise ferroelectric hysteresis was observed. As a result, a low *SS* value near 60 mV/dec could be achieved without compromising the overall performance of AlGaN/GaN MOS-HEMTs.

## 2. Experimental

**Figure 1:**
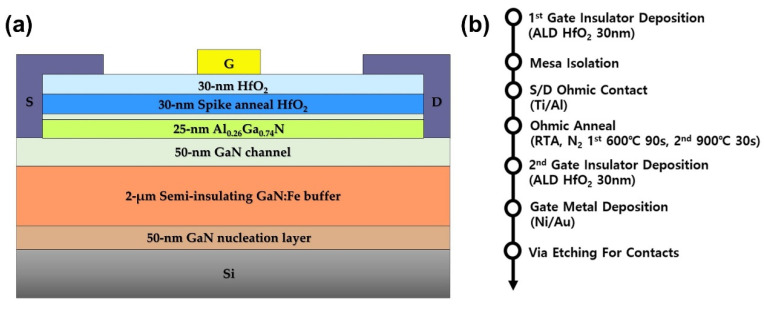
(**a**) A cross-sectional schematic of fabricated MOS-HEMTs with spike RTA HfO_2_ gate dielectric. (**b**) Summarized process steps.

Figure 1a shows the cross-sectional schematic of fabricated MOS-HEMTs with HfO_2_ gate dielectric. The AlGaN/GaN structure was grown by metal–organic chemical vapor deposition on a sapphire substrate, consisting of a 50-nm GaN nucleation layer, a 2 µm semi-insulating GaN:Fe buffer, a 50 nm undoped GaN channel, 1 nm AlN interlayer, a 25 nm Al_0_._25_Ga_0_._75_N barrier, and a 3 nm GaN capping layer. The room-temperature Hall mobility and sheet carrier concentration were 2200 cm^2^/Vs and 7.9 × 10^12^ cm^−2^, respectively. After cleaning the sample using acetone and 2-isopropanol (IPA) for 10 min each in an ultrasonicator, a 30 nm thick (1Å/cycle) HfO_2_ dielectric layer was deposited by atomic layer deposition (ALD) at the stage temperature of 350 °C. For the ALD process of HfO_2_, tetrakis (ethylmethylamino) hafnium (TEMAH) was the precursor and ozone was the reactant. A mesa was formed using Cl_2_/BCl_3_ inductively-coupled plasma reactive ion etcher. After the mesa formation, a Ti/Al (75/180nm) stack was deposited by and an e-beam evaporator for the ohmic source (S) and drain (D) contacts. These contacts and the gate oxide were spike-annealed at 600 °C for 90 sec and 900 °C for 30 sec using a rapid thermal annealing (RTA) process in the N_2_ atmosphere [14]. The ramp-up rates were 30 °C/sec from room temperature to 600 °C and 90 °C/sec from 600 to 900 °C. The cooling rate was 150 °C/min. The purpose of the first annealing step is twofold. While it is a temperature stabilization step before a spike annealing step, it is also to improve the ohmic contact and induce a phase transition within HfO_2_ for ferroelectricity. The second annealing step is solely to lower the contact resistance. The annealing had to be shortened to protect the ferroelectric phase in HfO_2_ and maintain the abrupt interface. As a result, the temperature had to be raised above normal ohmic contact annealing temperature (850 °C). To minimize the gate leakage through the grain boundary between the crystallized HfO_2_, a 30 nm thick amorphous HfO_2_ dielectric layer was deposited under the same ALD condition [15,16]. Finally, Ni/Au (20/50 nm) contact was formed on the gate dielectric. Figure 1b shows summarized process steps.

The electrical performance of fabricated MOS-HEMT was conducted via DC and PUND measurement. A 4200A-SCS semiconductor parameter analyzer ( Solon, OH, USA) was used for I-V measurements. PMU-4225 (Solon, OH, USA) with a remote pulse measure unit is used to perform PUND measurements. Additional source meter and DC power supply (Keithley 2410 and 2220G, Solon, OH, USA) were set up to apply a high voltage and measure breakdown voltage. The structural and interfacial analyses of the oxide-semiconductor structure were performed using TEM.

## 3. Results and Discussion

**Figure 2:**
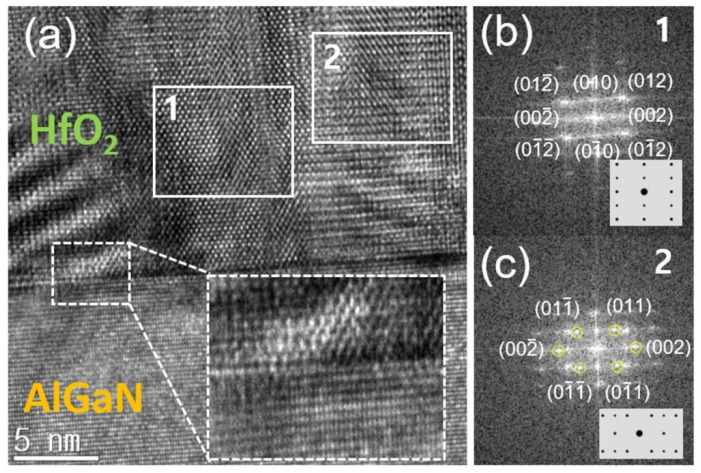
(**a**) A cross-sectional HR-TEM image of the HfO_2_/AlGaN with the inset showing the magnified image at the interface region. TEM diffraction patterns (zone axis = [100]) from the regions (**b**) 1 and (**c**) 2 with the insets showing the simulated diffractions patterns of the orthorhombic (Pca2_1_) and tetragonal (P42/nmc) phased for regions 1 and 2, respectively.

**Figure 3:**
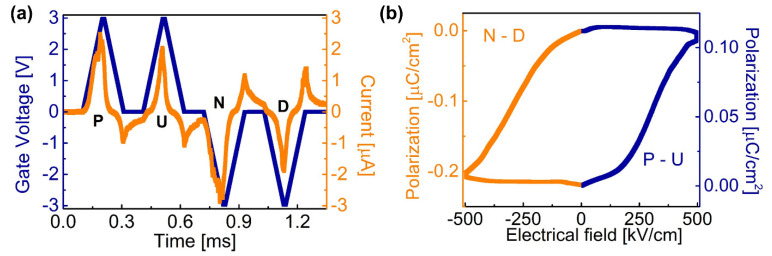
(**a**) PUND measurements using +3 V gate voltage pulses on the fabricated AlGaN MOSCAP. (**b**) Gate voltage-dependent polarization with the orange line from the N-D half polarization curve and navy line from P-U half polarization curve.

It has been reported that HfO_2_ can be ferroelectric when doped with elements including Al, Gd, Si, Zr, etc. [17]. The origin of ferroelectricity in the doped HfO_2_ film has been associated with a non-centrosymmetric orthorhombic phase (Pca2_1_) [18]. Undoped HfO_2_ can also display ferroelectric characteristics when an orthorhombic phase is present [19]. Therefore, regardless of doping, the key to the ferroelectric behavior of HfO_2_ is strongly related to an orthorhombic phase within the matrix. Figure 2 shows the cross-sectional TEM image of HfO_2_ on AlGaN after the second annealing at 900 °C for 30 sec. Given that the phase transition of HfO_2_ starts at ~500 °C [19], the orthorhombic HfO_2_ was likely formed during the fast short annealing step at 600 °C. While undesirable for the HfO_2_ layer, a 900 °C annealing step is necessary for the ohmic contacts. According to the cross-sectional TEM image, there was no evidence that the second contact annealing degraded the HfO_2_/AlGaN interface. The atomically abrupt interface between HfO_2_ and AlGaN was observed as indicated by the white dotted box in Figure 2a. The diffraction patterns (zone axis = [100]) from regions 1 and 2 are shown in Figure 2b,c, respectively. Based on the comparison between the measured and simulated patterns (the insets in Figure 2b,c), one can see regions 1 and 2 correspond to the orthorhombic (space group: Pc2_1_) and tetragonal (space group: P42/nmc) phase, respectively. In the case of region 2, however, note that the diffraction pattern is a mixture of both phases. This non-centrosymmetric orthorhombic phase within the HfO_2_ film is likely the origin of the gate voltage-dependent polarization switching shown in Figure 3. Note that the polarization charge measured from our samples is relatively low in comparison with an undoped HfO_2_ layer sandwiched in between TiN. This is likely because of the absence of a capping layer that suppresses the phase transition from the ferroelectric to paraelectric phase [20]. Although the ferroelectric phase and the abrupt HfO_2_/AlGaN interface were confirmed by TEM, the 2-step annealing process without a capping layer likely weakened the ferroelectric strength. Details of the ferroelectric property are discussed below.

**Figure 4:**
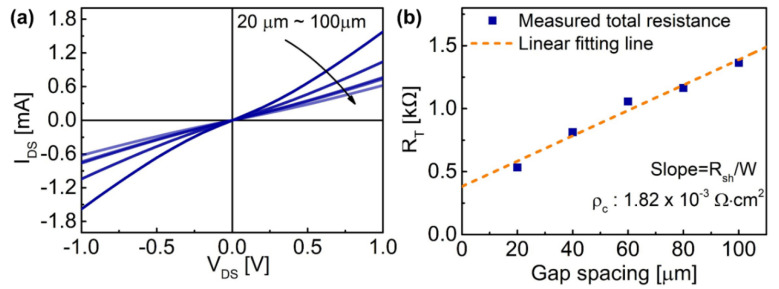
(**a**) The *I*_DS_ vs. *V*_DS_ curve measured at TLM pattern with different gap spacing from 20 µm to 100 µm. (**b**) The total resistance and contact resistivity which were calculated from Figure 2a.

Due to the unconventional S/D metal scheme adopted in our device structure, transfer length method (TLM) measurements were conducted to characterize the contact resistance of the S/D metal scheme in our device structure [21]. The total resistance (R) for variable gap spacings from 20 µm to 100 µm was calculated from the *I*_DS_ vs. *V*_DS_ curves in Figure 4a. After the measured R over the gap spacing is plotted, the contact resistance (R_c_) and specific contact resistivity (*ρ*_c_) were extracted as 19.1 Ω·mm and 1.8 × 10^−3^ Ω·cm^2^, respectively through a linear fit on the results as shown in Figure 4b. The relatively high contact resistance is attributed to the contact structure, which is contacted to the 2DEG through the corner of the sidewall, not through AlGaN/GaN layer. However, the sheet resistance (R_sh_) is extracted as 374 ohm/□, which is close to the sheet resistance measured by the Van der Pauw method. Therefore, the relatively high contact resistance does not seem to interfere with the ferroelectric effect of the spike-annealed HfO_2_ layer on 2DEG.

To examine the ferroelectricity of the HfO_2_ layer, the P-V PUND measurements were performed on the fabricated HfO_2_/AlGaN MOSCAP. PUND is a method that utilizes the displacement current (D) produced across a ferroelectric film. Before applying the positive switching voltage, D equals 2P_S_ + ϵE, where P_S_ is a spontaneous polarization field, ϵ is a dielectric constant of an insulator, and E is an applied field. The second positive pulse is a non-switching pulse that makes D simply ϵE. Therefore, the spontaneous polarization charge induced by a ferroelectric film is given by the difference in D between the first and second pulses. When it comes to measuring the actual polarization charge in a MOSCAP, a typical metal–insulator–metal (MIM) capacitor is not ideal. The measured polarization charge from a MIM is often different from the actual polarization charge in a real MOS device due to the surface potential of the semiconductor and trapped-charge induced screening of polarization. The leakage current can also be affected by the difference in the barrier height between a MOSCAP and MIM. Furthermore, the crystallinity of a ferroelectric film deposited on metal is different from the one deposited on the semiconductor. As the ferroelectric property is dependent on the domains, crystallinity plays a critical role in the magnitude of spontaneous polarization. Therefore, this work followed the method suggested in [24] to perform PUND on our MOSCAP [22,23,24]. Figure 3a shows the current (orange) measured in response to the PUND pulses; P and N voltage pulses correspond to switching polarization. The measured current for N and D pulses is larger than that of P and U, which leads to different magnitudes for each polarization half-loop as calculated in Figure 3b. Moreover, the amount of switched charge for the N pulse is larger compared with the P pulse; the estimated induced charge ranges from 0.11 to 0.22 µC/cm^2^. Considering the amount of 2DEG is ~1.3 µC/cm^2^ (carrier concentration = 7.9 × 10^12^/cm^2^), these additional charges induced by the polarization are expected to increase the carrier density of 2DEG by ~10% and thereby lower the subthreshold swing of HEMTs.

**Figure 5:**
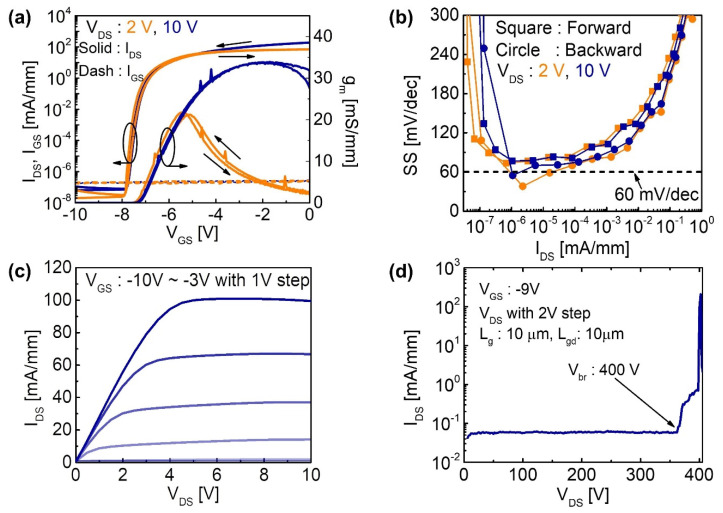
(**a**) *I*_DS_-*V*_GS_ and transconductance curves with *V*_DS_ at 2 V (orange) and 10 V (navy). (**b**) Measured subthreshold slope as a function *I*_DS_ in forward (square) and backward (circle) directions with *V*_DS_ = 2 (orange) and 10 V (navy). (**c**) *I*_DS_-*V*_DS_ curves at various *V*_GS_. (**d**) Off-state showing breakdown at ~400 V.

Figure 5 shows the electrical characteristics of a representative device with *L*_G_ of 10 µm, channel length (*L*_DS_) of 30 µm, and channel width (*W*) of 100 µm. Figure 5a shows the transfer curves (*I*_DS_–*V*_GS_) normalized with *W* for *V*_DS_ = 2 and 10 V. The device exhibits a high on/off ratio of >10^9^ and a negligible hysteresis (Δ*V*) related to the interfacial traps [25]. Δ*V* is approximately 160 and 70 mV at *V*_DS_ of 2 and 10 V, respectively. *I*_DS_ is lower than state-of-the-art devices due to the long channel length and unoptimized device structure. However, Figure 5b illustrates the subthreshold slope (*SS*) close to 60 mV/dec during the transient owing to the abrupt HfO_2_ (with spike RTA)/GaN interface confirmed by TEM analyses. The *SS* of 73.6 and 58.9 mV/dec is calculated from

(1) $$S=\frac{dV_{GS}}{d\left(log_{10}I_{DS}\right)}$$

under forward and backward *V*_GS_ sweeps, respectively, with 0.05 V/step and *V*_DS_ = 2 V. In addition to the TEM analysis in Figure 2 and small Δ*V*, the interface trap density (*D*_it_) was estimated using the equation

(2) $$D_{it}=\frac{C_{i}}{q^{2}}\left(\frac{qSS}{kTln10}-1\right)$$

The obtained *D*_it_ of 8.64 × 10^11^ eV^−1^ cm^−2^ is comparable to *D*_it_ of more conventional MOS-HEMTs such as the ones with Al_2_O_3_. Therefore, the spike-annealing process does not seem to degrade the interface. Considering the similar *D*_it_ and material quality of our devices to the others, the lower *SS* of our devices is likely to be related to the weak ferroelectricity of the undoped HfO_2_ layer. Furthermore, note that even lower *SS* was observed during the backward sweep due to the ferroelectric dipoles within the HfO_2_ aligned toward the direction of HEMTs dipoles [26]. Figure 5c shows negligible current crowding and therefore good ohmic contacts. At *V*_GS_ = −3 V, however, self-heating of the device led to the lowering of *I*_DS_ in the saturation region. As shown by Figure 5d, the off-state breakdown of the device (*L*_GD_ = 10 µm) occurs at *V*_DS_ of ~400 V with *V*_GS_ = −9 V. The observed off-state *I*_DS_ was a few orders of magnitude higher than other measured I-V results in this work due to the high voltage measurement set-up.

**Figure 6:**
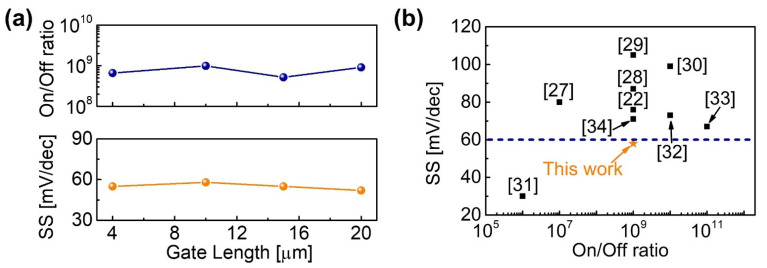
(**a**) Measured on/off ratio and *SS* as a function of the gate length. (**b**) Comparison of *SS* and on/off ratio values from various AlGaN/GaN MOS-HEMTs with a different gate dielectric as indicated.

Figure 6a shows the on/off ratio and *SS* values as a function of *L*_G_. A high on/off ratio of >10^9^ was maintained while exhibiting steep *SS* below 60 mV/dec under backward *V*_GS_ sweep. Figure 6b summarizes the reported *SS* and on/off ratio. Our MOS-HEMTs (*L*_G_ = 10 µm) show a relatively high on/off ratio of >10^9^ with near-ideal *SS* of 60 mV/dec. This study strongly suggests the spike annealing of HfO_2_ at 600 °C can induce ferroelectricity while maintaining the pristine HfO_2_/GaN interface with low *D*_it_.

## 4. Conclusions

In summary, we investigated the impact of undoped HfO_2_-based ferroelectric gate dielectric on the device performance of AlGaN/GaN HEMTs. The spike annealing process at 600 °C induced the orthorhombic phase, which is likely the origin of observed ferroelectric behavior. TEM analysis confirmed the atomically abrupt HfO_2_/nitride interface with no interlayer and an overall *D*_it_ of 8.64 × 10^11^ eV^−1^ cm^−2^ was estimated from the *SS*. The combination of the enhanced ferroelectricity and high interfacial quality led to *SS* close to 60 mV/dec and on/off ratio>10^9^. These results show that the HfO_2_-based ferroelectric gate can improve the efficiency of AlGaN/GaN MOS-HEMTs without compromising the overall performance of HEMTs.

## Data Availability

The data presented in this study are available on request from thecorresponding author.
